# Silver Nanoparticles against Foodborne Bacteria. Effects at Intestinal Level and Health Limitations

**DOI:** 10.3390/microorganisms8010132

**Published:** 2020-01-17

**Authors:** Irene Zorraquín-Peña, Carolina Cueva, Begoña Bartolomé, M. Victoria Moreno-Arribas

**Affiliations:** Institute of Food Science Research (CIAL), CSIC-UAM, C/Nicolás Cabrera 9, Campus de Cantoblanco, 28049 Madrid, Spain; irene.zorraquin@csic.es (I.Z.-P.); carolina.cueva@csic.es (C.C.); b.bartolome@csic.es (B.B.)

**Keywords:** foodborne antimicrobials, silver nanoparticles, gut and microbiota, health

## Abstract

Foodborne diseases are one of the factors that endanger the health of consumers, especially in people at risk of exclusion and in developing countries. The continuing search for effective antimicrobials to be used in the food industry has resulted in the emergence of nanotechnology in this area. Silver nanoparticles (Ag-NPs) are the nanomaterial with the best antimicrobial activity and therefore, with great potential of application in food processing and packing. However, possible health effects must be properly addressed to ensure food safety. This review presents a detailed description on the main applications of Ag-NPs as antimicrobial agents for food control, as well as the current legislation concerning these materials. Current knowledge about the impact of the dietary exposure to Ag-NPs in human health with special emphasis on the changes that nanoparticles undergo after passing through the gastrointestinal tract and how they alter the oral and gut microbiota, is also summarized. It is concluded that given their potential and wide properties against foodborne pathogens, research in Ag-NPs is of great interest but is not exempt from difficulties that must be resolved in order to certify the safety of their use.

## 1. Introduction

It is estimated that there are 600 million cases of foodborne illnesses and 420,000 deaths annually worldwide. Unsafe foods are a risk to human health and countries’ economy and mainly affect people at risk of exclusion, migrants and population under conflicts. The majority of foodborne diseases are related to pathogenic bacteria belonging to the genera *Salmonella*, *Listeria*, *Escherichia*, *Clostridium* and *Campylobacter*. Microbial contamination of food can occur at different stages of the process, such as harvesting, slaughtering, processing and distribution (“farm to fork”) and can be caused by environmental contamination, such as water, soil or air [1]. The most common symptoms of foodborne diseases are gastrointestinal, such as diarrhea, but other consequences may be kidney and liver failure, brain and neural disorders, reactive arthritis and others. These diseases can be more severe in children, pregnant women, the elderly and those with a weakened immune system [2]. Traditional techniques such as salting, drying, freezing or fermentation are applied to extend the shelf life of food products, but there may be risk of recontamination. Therefore, there is a continuous need for antimicrobial agents that act in both food processing (preservation) and packaging (safety) stages [3].

In recent years, nanotechnology has experienced a noticeable rise in its applications, from agri-food to biotechnology, going through the engineering, cosmetic and textile industry. It can be considered a technological revolution [4]. Focusing on the field of food and health, nanotechnology is used in drug delivery system and nutrient release systems (nanoencapsulation), increasing the rate of recognition of disease symptoms and providing rapid treatments. It can also be applied to crops in the form of fertilizers and nanoscale additives or create nanoscale sensors to detect chemical, viral or bacterial contamination. In the case of food processing, it is a still emerging but promising technology [5].

Nanomaterials can be natural, accidental or manufactured and can be constituted by loose particles, aggregates or agglomerate in the form of nanoparticles, nanotubes, nanowires, nanofibers, and others. Of these, nanoparticles (NPs), wherein 50% or more of them in the numerical granulometry have one or more of the external dimensions between 1 and 100 nanometers, are possibly the most studied and the ones having more variety of sizes and shapes, which results in a large number of technological applications [6,7,8].

NPs are generally classified into organic and inorganic. Organic NPs incorporate carbon, whereas inorganic NPs incorporate metallic (Ag, Au, Cu), magnetic (Co, Fe, Ni), and/or semi-conductor components (ZnO, ZnS, CdS) [9]. Focusing our interest on silver nanoparticles (Ag-NPs), these have been widely used in medicine and biotechnology fields, due to their properties as antimicrobials. In this sense, numerous research studies have confirmed the effectiveness of Ag-NPs to inhibit the growth of pathogenic bacteria such as *Staphylococcus aureus*, *Streptococcus mutans*, *Streptococcus pyogenes*, *Escherichia coli* and *Proteus vulgaris* [10,11,12]. Interestingly, this activity has been also demonstrated using Ag-NPs obtained by ‘biological methods’ which are considered a great tool to reduce the negative effects associated with traditional nanoparticle synthesis commonly used in the laboratory [13]. In particular, two recent studies have shown the antimicrobial activity of Ag-NPs from apple pomace and from exopolysaccharides isolated from green microalgae against *E. coli* and *S. aureus* [14,15].

**Figure 1:**
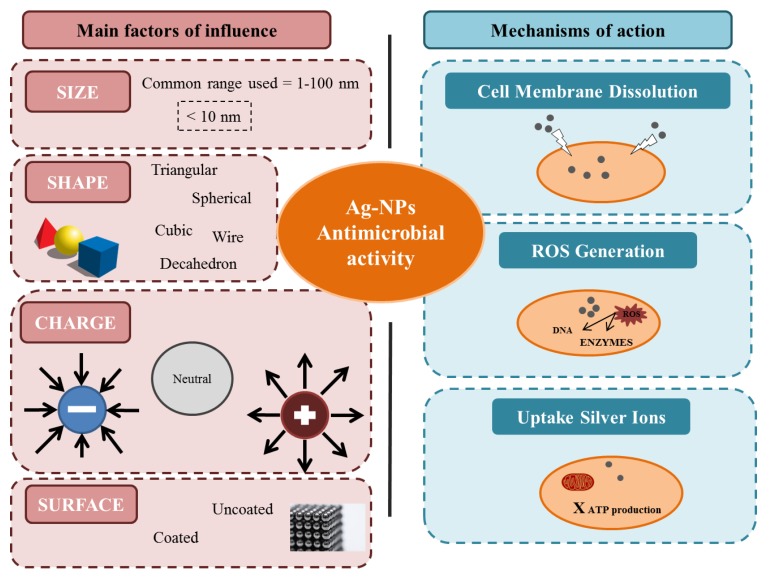
Main factors of influence and hypothetical mechanisms for the antimicrobial activity of silver nanoparticles.

Shape, size, surface and charge are highlighted as the factors that influence the antimicrobial properties of Ag-NPs (Figure 1). Regarding shape (i.e., triangular, decahedron, spherical, cubic, platelet, among others), the spherical and the triangular forms seem to lead to higher antimicrobial activity [16,17,18]. Size is one of the most important factors when synthesizing nanoparticles, 1 to 30 nm being the most widely used range. Many studies have shown the size-dependent antibacterial activity of Ag-NPs [19,20,21,22]. Concerning the nanoparticle surface, it may be modified through the addition of coating agents, such as polymers (chitosan, polyethyleneimine, polyethylene glycol, polygamma glutamic acid), proteins (milk casein, bovine serum albumin, human serum albumin), antioxidants (glutathione) and/or polyvalent anion salts. Finally, the charge of the Ag-NPs determines their interaction with biological environments and its cellular uptake, which leads to a modulation of its antibacterial activity. Moreover, the antimicrobial activity of silver nanoparticles is bacteria strain- and cell wall structure-dependent [23].

The mechanisms of action by which Ag-NPs exert their antimicrobial effects are not completely clear, but two main hypothesis have been proposed: (*i*) a direct interaction of the nanoparticle with the cell membrane, and (*ii*) the release of ionic silver [24]. In the first hypothesis, the Ag-NPs would be adhered to the cell membrane via electrostatic attractions between the positive charges of the nanoparticles and the negative charges of the cells [25] or via the interaction of the nanoparticles into the sulfur and phosphorylated proteins present in the cell wall [26]. In any case, the interaction of the Ag-NPs with the cell membrane would produce its partial dissolution (Figure 1). In the second hypothesis, the Ag-NPs would enter into the cell and lead to a release of silver ions and the subsequent increase of reactive oxygen species (ROS) that would damage the enzymes involved in the cellular oxidation-reduction respiratory process and be finally responsible for cell death [16] (Figure 1). The two hypotheses could occur together as it has been showed that after interaction of the nanoparticle with the cell membrane, an internalization step takes place. In turn, this process can be affected by the nanoparticle charge [27]. Despite the antimicrobial effectiveness, some bacterial resistance against silver nanoparticles has been reported. Mechanisms such as negative regulation of porins, chromosomal resistance genes or plasmids with resistance genes have been proposed. However, this is still a field under study and more information to clarify this point at the frame of the food industry is clearly needed [24,28].

On the other hand, the increased incorporation of silver nanoparticles into consumer products makes it essential to address their potential risk for human health. Nevertheless, there is still a lack of knowledge about their specific aspects of the intestinal uptake of silver nanoparticles [5]. The oral route of exposure has been poorly explored, despite the incorporation of such nanoparticles into packaging in contact with foods. After their ingestion, these nanoparticles pass through the digestive tract, where they may undergo physicochemical transformations, with consequences for the luminal environment, before crossing the epithelial barrier to reach the systemic compartment. Therefore, Ag-NPs toxicity and in particular, their effects at the gut level, are major concerns in the use and development of these nanomaterials.

This review presents a detailed description on the main applications of silver nanoparticles as antimicrobial agents for food control, as well as the current legislation concerning these materials. In addition, we summarize current knowledge about the impact of the dietary exposure to silver nanoparticles in human health with special emphasis in the gastrointestinal environment and microbiota, and highlight the areas where information is lacking. Finally, conclusions and future directions about both topics are summarized.

## 2. Applications of Antimicrobial Silver Nanoparticles in the Food Industry

Microbial food spoilage is a major global concern that can reduce the shelf life of food while increasing the risk of foodborne diseases. In this framework, the use of well-known potent antimicrobial agents such as silver nanoparticles constitutes an interesting approach. An overview of the effectiveness of silver nanoparticles to inhibit the growth of different microorganisms is shown in Table 1.

**Figure 2:**
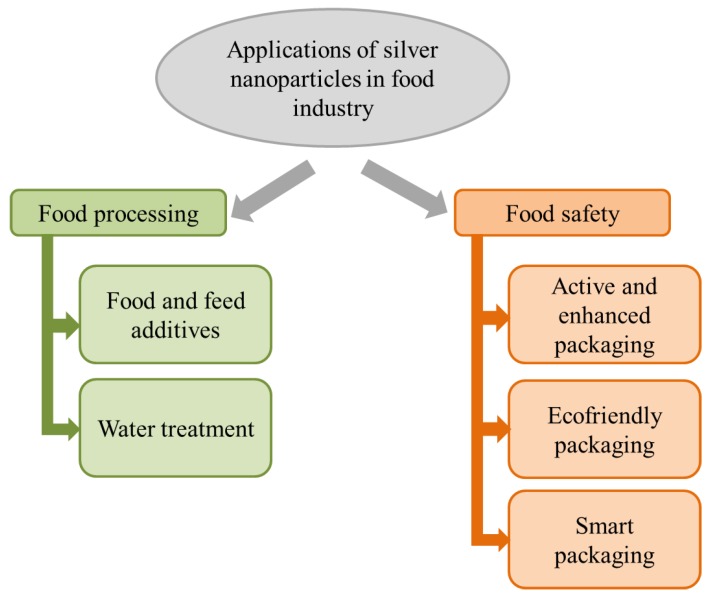
Classification of the use of Ag-NPs as antimicrobials in the food industry (adapted from Singh et al. [39]).

Among other relevant results, Silvan et al. [33] demonstrated the antibacterial effect of Ag-NPs against multi-drug resistant (MDR) *Campylobacter* strains isolated from the chicken food chain and clinical patients. In another study, nanoparticles synthesized through *Forsythia suspensa* fruit water extract showed antibacterial activities against the most common foodborne pathogens, including *Listeria monocytogenes*, *Vibrio parahaemolyticus*, *Escherichia coli* O157:H7 and *Salmonella typhimurium* [36]. Similarly, the Ag-NPs synthesized from jack fruit seeds showed an antibacterial effect against *E. coli* and *S. typhimurium* [35]. The toxic effect of Ag-NPs synthesized using a bacterial exopolysaccharide as a reducing and stabilizing agent against various food pathogens (*L. monocytogenes*, *Aspergillus* spp. and *Penicillum* spp.) was also demonstrated [31]. Based on these promising results and in order to improve the shelf life and safety of food, there are various food preservation and safety strategies in which Ag-NPs have been employed (or proposed to be employed) in the food industry (Figure 2). Specifically, in this section, we report on the last studies assessing the use of silver nanoparticles in food processing and food packaging, and also the current regulation about it.

### 2.1. Food Processing (Preservation)

As feed additives, Ag-NPs has shown to be effective in the reduction of potentially pathogenic organisms such as *E. coli* and *Clostridium perfringens* [40,41,42,43], which could reduce the use of antibiotics in livestock [43]. Additionally, some Ag-NPs have also showed effective antiparasitic activity [44,45,46]. 

Ag-NPs have also been successfully applied in water treatment by incorporating them to filters with foam or by impregnating ultrafiltration membranes [29,30]. Although the investigation of Ag-NPs as a food additive is not very widespread, attractive attempts have been made to replace the use of sulfur dioxide by the use of antimicrobial nanoparticles. This is the case in the wine industry. For example, the effectiveness of a colloidal silver complex of a size < 1 nm was studied, managing to control the growth of lactic acid bacteria [47,48]. Another study confirmed the antimicrobial activity of two coated Ag-NPs against lactic acid bacteria and other microorganisms such as *S. aureus* and *E. coli*, with potential application in winemaking [49].

### 2.2. Food Packaging (Safety)

Food packaging is one of the areas where nanoparticles research and use is most relevant. The need of protection against foodborne diseases and the requirement of consumers to extend the useful life of the products urged the development of antimicrobial food packaging, special packaging that releases active biocide substances in order to improve the quality of the food [50]. The use of natural substances, such as green tea and chilto extracts and essential oils in packaging materials has already been investigated [51,52,53] but the use of Ag-NPs would be a more effective alternative because their antimicrobial activity is greater than phytochemicals. Nanotechnology in food packaging can be divided into three categories: (*i*) active packaging, (*ii*) ecofriendly packaging, and (*iii*) smart packaging, although packaging combinations are also possible (i.e., active and ecofriendly packing). In *active packaging,* the silver nanoparticles interact directly with the food or the environment polymer matrix which can be a non-degradable polymeric film such as polyethylene (PE), polyvinyl chloride (PVC) and ethylene vinyl alcohol (EVOH) or a biodegradable edible coating film made by a polymer or a stabilizing agent (ecofriendly packaging). In addition, Ag-NPs offer a good stability and slow release rates of silver ions in stored foods which makes them suitable candidates to be used in food packaging [54]. In line with this, Yu et al. [38] demonstrated the antibacterial effect of a material composed of Ag-NPs and cellulose nanofibrils against *E. coli* and *L. monocytogenes*. Similarly, silver nanoparticles immobilized with laponite showed a good growth inhibitory activity against *E. coli*, *S. aureus*, *A. niger* and *P. citrinum* [34]. Similar effects of silver nanoparticles against chicken meat (breasts and sausages) were found. The bacterial growth of *S. aureus*, *S. typhimurium* decreased, although there were also increases in cadaverine and thiamine [32,37]. On the other hand, the protective effect of silver nanoparticles in long-term packaging of nuts has also been demonstrated. The 3% silver package achieved a significant reduction in the presence of mold and coliforms and also achieved an antioxidant effect. Finally, the silver nanoparticles had a significant effect on increasing the shelf life of nuts [55].

At the framework of food safety, smart packaging, that is, packing with biosensors for the detection of pathogens represents a novel approach for food preservation, although still under development. The operation mode is based on the union or reaction of biological components with target species (microorganisms, toxins, etc.) and the transformation into detectable signals, which leads to the rapid detection of food contaminants [56]. Examples can be found in studies such as Abbaspour et al. [57] which described a selective sandwich biosensor for the detection of *S. aureus*. Combination of fluorescent carbon points (CDF) with silver nanoparticles has been reported for the detection and elimination of bacteria such as *E. coli* and *S. aureus* at low concentrations [58]. In the same way, the conjugated polyelectrolytes (CPs)–silver nanostructure pair has showed a high detection power against *E. coli* [59].

### 2.3. Regulation about Silver Nanoparticles Use in Foods and Food Industry Packaging (Safety)

The panel of the European Food Safety Agency (EFSA) on food additives and sources of nutrients added to food determined that there is insufficient information on Ag to assess its risk and, therefore, in the European Union (EU), Ag-NPs are not allowed in food supplements or food packaging unless authorized. EFSA has also provided Ag migration limits from the packaging (<0.05 mg/L in water and <0.05 mg/kg in food [60,61]. Therefore, manufacturers must carry out migration evaluations as well as genotoxicity, absorption, distribution, metabolism and *in vitro* excretion tests [60,61]. With all this information, EFSA will carry out a risk assessment of the specific case to determine if that package can be marketed or not. To date there are no known products that have been approved. On the other hand, in November 2015, Regulation (EU) 2015/2283 of the European Parliament and the European Council on new foods was approved. In this regulation, it appears the definition of “artificial nanomaterial” to include, within this new category (“novel foods”), all the foods that consist or contain artificial nanomaterials [62]. In spite of this, Ag-NPs still do not appear in the legislation of allowed food additives or in the materials in contact with food. Otherwise, in the United States, these regulations are influenced by the existing regulatory restrictions on the release of silver to the environment and are the responsibility of three agencies: the Environmental Protection Agency (EPA), the Food and Drug Administration (FDA) and the agency of the Institute National Occupational Safety and Health (NIOSH). The FDA published a guide for the use of nanotechnology in food or materials in contact with them and recommended that manufacturers study and prepare a toxicological profile for each container with nanomaterials [63,64].

As mentioned above, one of the problems of using these nanoparticles in food packaging is silver migration. Echegoyén and Nerín [65] conducted an analysis of the form of silver migration, whether ions or particles, into food simulants. They demonstrated that silver migrated to food and was dependent on food and warming, with acidic foods and oven heating presenting a higher migration. However, in their study, they found that Ag migration is well below the maximum migration limits established by European Union legislation. However, other studies did not observe any temperature or time-dependent increase in the migration of Ag packaged foods [66]. Gallocchio et al. [67] tested a container with Ag-NPs to store chicken breasts and did not observe that the silver content of the breasts was higher than that allowed by the EU.

## 3. Impact of Dietary Exposure to Silver Nanoparticles in Health: Gut Nanotoxicology Effects

As the investigation into the application of nanotechnology in the food sector increases, the potential of nanotechnology in food science/industry also expands and consequently, so does the human exposure to these substances. In the case of antimicrobial silver nanoparticles with application in food industry, the subject of this review, the main human exposure source is through the oral-gastrointestinal tract [68]. The mean dietary exposure level of Ag-NPs is estimated at 70–90 μg/day [69]. After ingestion, the Ag-NPs come in contact with lumen of the oral cavity and esophagus. There is little published information on the absorption rate of particulates through the epithelium of these two compartments, probably due to both a low surface area and a short residence time for most food matrices [68]. After that, during the gastrointestinal digestion process in the stomach and small intestine, the interaction of Ag-NPs with biological fluids can lead to its agglomeration, aggregation, and dissolution [69,70,71,72,73]. In addition, silver nanoparticle absorption (transcellular and paracellular transport and vesicular phagocytosis) through the gastrointestinal tract epithelium could take place. Finally, the nanoparticles that escape the absorption process reach the colon where they could modulate the composition and/or activity of gut microbiota, affecting the production and toxicity of bacterial metabolites [69]. Part of the initial intake of nanoparticles could be extracted in feces. According to the anatomy of the gastrointestinal tract, several environments characterizdc by specific microbiota composition are found. Gut microbiota harbors more than 100,000 billion microorganisms, including bacteria, fungi, viruses, protozoa and archaea, with bacteria representing a majority. The dominant gut bacterial phyla are the *Firmicutes* (including *Clostridium*, *Enterococcus*, *Lactobacillus*, and *Ruminococcus* genera) and *Bacteroidetes* (including *Bacteroides* and *Prevotella* genera). These bacteria play an important role in the development and conservation of host health. Gut microbes play a role in human physiology through several mechanisms, including their contribution to nutrient and xenobiotic metabolism (e.g., synthesis of vitamins, digestion of oligo, and polysaccharides, drugs, etc.) and to the regulation of immune and neurodendocrine functions. Some of these effects are mediated by products of bacterial metabolism, such as short-chain fatty acids (SCFA), including propionate, butyrate or acetate, which influence the gut barrier, the inflammatory tone and the metabolic homeostatic control in different tissues [74]. To date, little is known about the effect of nanoparticles on the intestinal microbiota, but what is known is that there are numerous factors that can produce an imbalance in the intestinal bacterial populations, like food, triggering certain diseases. That is why the investigation of the NPs-gut microbiota relationship is so important and should continue [68,69]. 

The physical and chemical transformations of Ag-NPs during the gastrointestinal digestion could involve modifications in their toxic effect. Despite the specific features of these particles and the differences among them, they all display a close relationship between physicochemical reactivity and bioavailability/biopersistence in the gastrointestinal tract. Recently, Mercier-Bonin et al. [68] and Bouwmeester et al. [72] discussed the potential impact of the luminal and gastrointestinal environment on nanomaterial properties and toxicity studies. In this section, with a specific focus on silver nanoparticles, we report *in vitro* and *in vivo* studies considering both local and systemic levels effects, with a particular emphasis on their impact on gut microbiota. 

### 3.1. In Vitro Studies: Static and Dynamic Gut Simulators and Epithelium Cell Models

Today, several *in vitro* models, from cell models to static and dynamic gastrointestinal models can be used alone or in combination for the study of Ag-NPs toxicity. As mentioned above, concentration/dose is a very important factor for the use of nanoparticles as an antimicrobial agent in the food field. In general, cytotoxicity of Ag-NPs is concentration-dependent. Moreover, depending on the cell type, silver nanoparticles cytotoxicity varies notably, and this should be taken into consideration for their application in consumer products [75]. As said above in relation to their antimicrobial activity, size, shape, charge and surface are also factors that affect the cytotoxicity of these nanoparticles. Ag-NPs’ security depends on their state as they can form aggregates during their synthesis and use due to surface charge or they are covered by a high viscosity substance or suspended in a high viscosity environment. It has been shown that coated silver nanoparticles have lower cytotoxic due to the stabilization effect of the coating, which in turn, depends on the coating material and the thickness of the layer [76,77].

Different studies have evaluated the cytotoxic effect of silver nanoparticles in various human cell lines trying to understand the possible risks after exposure or ingestion (Table 2). However, today there are not many studies that evaluated the effect of silver nanoparticles in the oral cavity and the evaluation of the effect of these nanoparticles on oral microbiota is even more limited [68]. In one of these studies, it was found that Ag-NPs increased oxidative stress, inflammation and apoptosis in the human gingival fibroblast cell line (CRL-2014) [78]. Likewise, Niska et al. [79] observed that Ag-NP induced cell death in a concentration-dependent manner, not being toxic until concentrations greater than 40 μg/mL on human gingival fibroblasts (HGF-1). On the other hand, Hernández-Sierra et al. [80] studied the effect of Ag-NPs of different sizes of periodontal fibroblasts extracted from volunteers. They concluded that only nanoparticles with a size smaller than 20 nm increased the cytotoxicity of fibroblasts. Another study with human periodontal fibroblasts, specifically with the cell line HPLF, found that nanoparticles at low concentrations (≤16 μg/mL) had little influence on proliferation and cell cycle, while at high concentrations (32 and 64 μg/mL), they inhibited cell proliferation and significantly changed morphology [81]. The effect of Ag-NPs on oral bacteria has also been evaluated, with bacteria of the genus *Streptococcus* being more sensitive to them [82]. In another work, it was observed how the MIC and MBC of the silver nanoparticles was between 100 and 250 µg/mL for peri-implantitis pathogens [83]. On the other hand, Lu et al. [19] reported a MIC range between 25 and 50 µg/mL and this could be due to the smaller size of the nanoparticles used.

Unlike what happens with the oral cavity, there are numerous *in vitro* investigations on the effect of silver nanoparticles in the intestine (Table 2). It was observed that the intake of Ag-NPs within a food matrix increased its absorption by colon epithelial cells, the opposite being the case when ingested without food. This shows us the ease with which nanoparticles can reach our intestines due to their consumption along with food [84]. The toxicity difference between digested and undigested silver nanoparticles was also studied. It was possible to verify how the undigested ones were mostly captured by the cellular model Caco-2/HT29-MTX [88]. In the study of Silvan et al. [33], exposure of GSH-Ag NPs to epithelial cells (HT-29, Caco-2 and CCD-18) showed a dose-dependent cytotoxic effect and no significant cytotoxicity occurred until concentrations of 4.93 μg/mL. This is supported by other works in which the toxicity of silver nanoparticles is usually in the range of 10 to 100 μg [93]. It was observed in the work of Vila et al. [87] that the exposure of small-sized Ag-NPs (≈ 8 nm) at a concentration of 100 µg/mL only reached 20% cytotoxicity in Caco-2 cells. It was also shown that cell integrity was not altered using concentrations below 50 µg/mL. 

The toxicity of these nanoparticles has not only been studied on oral and intestinal cell lines. There is a study in which non-cytotoxic doses of Ag-NPs were used against the HepG2 cell line. Moreover, at low doses (2 and 4 mg/L), Ag-NPs presented “hormesis” effects by accelerating cell proliferation and an activation of mitogen-activated protein kinase (MAPK) [91]. On the other hand, Khorrami et al. [92] described a cytotoxicity level of 70%, at concentrations between 10 and 60 µg/mL, on the MCF-7 breast cancer cell line, while for the L-929 cell line (non-carcinogenic), it was only 15%. In another study, the toxic effect of Ag-NPs on the MCF-7 cell line was also evaluated. Cellular cytotoxicity was observed from a nanoparticle concentration of 10 µg/mL [90]. This is opening the door to the use of this nanomaterial against cancer cells and therefore, to be a possible cancer therapy, alone or in combination with other existing methods [86,94]. Other studies reported that Ag-NPs may interact with the cerebral microvasculature producing a proinflammatory cascade in rat brain microvessel endothelial cells, as well as that larger NPs were less toxic, and blood–brain barrier (BBB) dysfunction and astrocyte swelling causing neuronal degeneration [89,95]. 

In reference to static models of gastrointestinal digestion (Table 3), there is one study that showed that Ag-NPs with a size of 60 nm and a concentration of 10 mg/mL (1661 particles/mL) in the presence of proteins survived the extreme conditions of the digestion and reached the intestine [71]. This probably means that epithelial cells of the intestine would be exposed to these nanoparticles, causing cellular damage. On the contrary, in the absence of proteins, the fraction of NPs that reached the intestine was smaller [71]. In other works they also studied the effect of nanoparticles during the passage through the gastrointestinal tract. It was found that by contacting them with synthetic human stomach fluid, the Ag-NPs aggregated significantly and also released ionic silver that was physically associated with the aggregates of particles such as silver chloride. In addition, it was seen that NPs smaller than 10 nm were added to a greater extent than larger one [96]. It was also demonstrated that depending on the composition and pH, the morphology and the size of the Ag-NPs changed when passing through the different fluids (simulated saliva and gastric and intestinal fluids); in addition, there was only a low toxicity in a pilot study of reconstituted human tissues model [85]. When Ag-NPs interact with proteins, a corona is always formed and it decreases the entry of nanoparticles into cells and therefore, cellular toxicity decreases [97]. Gil-Sánchez et al. [74] evaluated the effect of static *in vitro* digestion on silver nanoparticles with two types of coating. It was observed that the glutathione-coated nanoparticles agglomerated less than those that had the polyethylene glycol coating and were less toxic to colon cells. Studying the changes of NPs in dynamic models is more limited. In the work of Cueva et al. [98], the dynamic gastrointestinal simulator simgi^®^ was used to digest Ag-NPs and study their effect on the colonic microbiota (Table 3). They did not observe changes in the bacterial composition or in the production of ammonium ions during the simulations, so it was concluded that Ag-NPs did not alter the composition and metabolic activity of the human intestinal microbiota. Another dynamic study showed that 90% of Ag-NPs were already dissolved by passing through the stomach and that many of the released ions bind to the food matrix. This results in less bioavailable ions and therefore, less toxicity (Table 3) [73]. 

A limited number of studies on the interaction of nanomaterials with the microbiome are available, most of them in rodents. In one *in vitro* study, it was observed that the Ag-NPs modified the *Firmicutes*/*Bacteroidetes* phylum ratio, increasing *Firmicutes* and decreasing *Bacteroidetes*. It was seen that the nanoparticles altered the intestinal microbiota as would a metabolic and inflammatory disease [100]. On the other hand, after exposure of silver nanoparticles (10 nm) to a concentration range of 0–100 µg/mL, a marked decrease in saturated fatty acids was observed, except in palmitic acid, which increased by 26–32%. The observation of these variations led to the sequencing of bacterial DNA. According to the results of Das et al. [99], Ag-NP ingestion, either deliberate or inadvertent, could have negative consequences on our intestinal microbiota, as evidenced by a significant decreasing of *Bacteroidetes* due to both ionic silver (AgCl; 25–200 mg/L) and nanosilver-mediated changes. 

### 3.2. In Vivo Studies: Animal and Human Trials

When conducting studies *in vivo*, five main types of models have been used: rats, mice, *Caenorhabditis elegans*, fish (zebrafish), *Drosophila melanogaster* and, in a lesser extent, human studies (Table 4). Each model has its advantages and limitations, but all provide a great deal of information that helps us to conclude facts. Within all these models, rats and mice may be the most used, but the one that generates the most interest is the human model, since it provides real data when it comes to human applications.

Regarding the findings with rat and mice, several studies have been carried out to evaluate the effect of these nanoparticles on the gastrointestinal tract. An abnormal mucus composition of the intestines of the animals was observed, as well as pigmentation of the villi and discharge of mucus granules [102,103,109]. In another study, it was discovered that Ag-NP damaged the microvilli of epithelial cells and intestinal glands in rats, thus decreasing the intestinal absorption of nutrients [104]. In the study by van den Brule et al. [111], by using Next Generation Sequencing (NGS), they observed how the intake of dietary doses Ag-NPs during 28 d did not significantly alter, in a dose-dependent manner, either the uniformity of the intestinal microbiota or populations in rats. But they could see an increase in the relationship between *Firmicutes* and *Bacteroidetes* phyla. Human and mouse gut microbiota are very similar at the phylum level, but not at the genera or species level; however, at least at the phylum level, these results could be extrapolated to humans. It was also discovered that the consumption of Ag-NPs modified the values of cholesterol and alkaline phosphatase in rats, which indicated that exposure to these nanoparticles could cause mild liver damage [103]. Silver nanoparticles are also easily able to cross the tight junction of the blood–brain barrier (BBB); therefore, they can be considered as neurotoxic. Rahman et al. [101] showed a neurotoxic effect induced by oxidative stress of Ag-NPs in three regions of the brain, including the caudate nucleus, the frontal cortex and the hippocampus of adult mice. In addition, another study showed that Ag-NPs produced neuronal degeneration and inflammation of astrocytes in the rat brain due to a low dose of exposure by oral and intragastric administration [105,106].

There are studies with other animal models like fishes. After exposure of fish at Ag-NPs concentrations of 2.5, 10, and 25 μg/L for 24 h, it was observed that the accumulation of silver in the brain was greater than in the liver and gills. In addition, fish that were exposed to the highest concentrations showed alterations in markers of oxidative stress [112]. In another study, various sizes of Ag-NPs coated with gum arabic (10, 40 and 100 nm) were used. Zebrafish embryos were exposed to various concentrations of these nanoparticles for 4 days and only an increase in lethality was observed with the 40 nm nanoparticles. This could be because of the retention of silver in the intestine depends on the particle size and the agglomerates [114]. In the same line is the work of Liu et al. [115], in which they demonstrated that the particle size is more influenced by the toxicity of Ag-NPs than the coating. Ag-NPs of small size (20 nm) and with citrate coating were more toxic and the toxic effect was greater in the intestine than in the gills or muscles. Merrifield et al. [113] showed, in adult zebrafish, that exposure to silver nanoparticles (500 mg/kg food) for 14 days had no effect on the richness and diversity of the microbiota. Similarly, Wilding et al. [107] found that the oral administration of silver nanoparticles of two different sizes (20 and 110 nm) and with two different coatings (PVP and citrate) for 28 days (10 mg/kg bw/day) did not change the diversity of the gut microbiome in mice. In another study, the effect of Ag-NPs in mouse models with inflammatory bowel disease was evaluated. A decrease in inflammation and a positive modulation of the gut microbiota could be observed [110]. By contrast, another study on rats fed twice-daily with oral silver nanoparticles for 13 weeks at various doses (9, 18 and 36 mg/kg bw/day) reported a general increase in the levels of Gram negative bacteria, and a decrease in the levels of *Firmicutes* [108]. It is important to note that there are differences between the human and zebrafish and rodent microbiome. Moreover, differences during gut transit and the interactions with the composition of the food matrix between animals and humans can affect nanomaterial properties in a different way during digestive transit and their putative effects. 

There are also studies with *Caenorhabditis elegans*. In one of them, it was observed how the reactive oxygen species in the nematode increased when exposed to *E. coli* contaminated with Ag-NPs. They also increased reproductive toxicity and neurotoxicity [117]. Moon et al. [118] showed that the presence of different silver nanomaterials (including nanoparticles) in the soil decreased the growth and reproduction of *C. elegans*. Similarly, in another study, the hereditary reproductive toxicity produced by Ag-NPs in *C. elegans* was demonstrated and it was observed that this toxicity contributed to inducing germline mutations [116,119].

Finally, another of the most used non-human models is *Drosophila melanogaster*. In one of the studies, the larvae were fed with silver nanoparticles, which were able to reach the intestinal barrier. This was demonstrated by analyzing the increase in intracellular ROS [123]. Reproductive toxicity was also evaluated in this model. It was observed that exposure of adult specimens to Ag-NPs significantly affected the ability to lay eggs along with a deteriorated ovarian growth [121,122]. In a study of acute and chronic exposure, it was observed that the effect of a solution of Ag-NPs at a concentration of 20 ug/mL 50% of the larvae did not end their development cycle. In addition, after chronic exposure to an Ag-Nps solution of 5 ug/mL, it was shown that after three generations, the flies adapted to silver, recovering the fecundity lost in the first three [120].

As can be seen in the aforementioned paragraphs, Ag-NPs have been shown to have toxic effects to both in *in vitro* and *in vivo* models; however, there is a limited number of studies that reported the impacts of Ag-NPs on human health. One of them is the one carried out by Munger et al. [124]. A total of 60 healthy subjects ingested nanoparticles at concentrations of 10 and 32 ppm (Ag-NPs size: 5–10 nm) for 14 days. No significant changes were detected in the morphology of heart, lungs and other organs, nor in the reactive oxygen species or in the generation of proinflammatory cytokines. Nor did significant changes in metabolic measures appear in the conditions studied. The authors stressed the need to evaluate the effects of longer-term exposure.

Because of the increased potential for consumer exposure to Ag NP, it appeared urgent to assess the possible impact on the gut microbiota and on human health. As reviewed, few studies have investigated this issue and none are conclusive. The differences of results between studies could be related to the techniques used to analyze the microbiota. Moreover, it is difficult to make a comparison between studies published today because different sizes, shapes and concentrations of nanoparticles have been used. As a suggestion, future experiments should consider validated standards to ensure more comparable results and thus, make more reliable conclusions. Moreover, the transfer of results from animals to humans could be improved with the use of “humanized” animals by inoculation of human gut microbiota as well as by investigations conducted with longer exposure durations to better mimic human exposure scenarios.

## 4. Conclusions and Future Perspectives

Nanotechnology and specifically, silver nanoparticles, have a promising future ahead in the field of food. Silver nanoparticles have demonstrated extensive antimicrobial activity against foodborne pathogens as well as great effectiveness when they are incorporated into different types of packaging. Today, most studies focusing on the use of Ag-NPs in packaging are at the laboratory level and in most countries, are not allowed. In the European Union, in particular, more data are necessary to define the regulation of their employment. Therefore, investigation of the use of nanoparticles as a food additive is needed, as well as the evaluation of their effect on consumer health, since there are no long-term studies that assess the real concerns of their consumption. Very few studies have focused on the relationships between nanoparticles and oral microbiota, and, in the same way, effects of silver nanoparticles on the composition of the intestinal microbiota and the consequences on their metabolic activity are largely unknown. The range of models and diverse experimental conditions, such as *in vitro*, *ex vivo* and *in vivo* approaches, animal models and control conditions, make it even more difficult to compare the results and draw final conclusions. A crucial aspect for *in vitro* studies is to take care to incorporate the changing physiochemical properties of silver nanoparticles during transit of the gastrointestinal tract in the study design. It is also necessary to continue studying the different types of silver nanoparticles including form, size distribution as well as dose and modes of administration/exposure of them to state detrimental effects on health. Finally, the difficulties involved in the evaluation *in vivo* of the effects of ingested nanoparticles in the gut, due to differences between species (rodents vs. humans), may also be highlighted. Probable variability between individuals, not only in terms of the composition, but also in terms of the functional metabolic properties of the microbiota, should also be taken into account along with host physiological characteristics and environmental factors. In conclusion, given their potential and wide properties against foodborne pathogens, research into silver nanoparticles is of great interest for the food industry but is not exempt from difficulties that must be resolved in order to certify the safety of their use.

## Figures and Tables

**Table 1:** Recompilation of studies about the antimicrobial effects of Ag-NPs against foodborne pathogens.

Ag-NPs Size	Ag-NPs Concentration	Gram (-) Pathogens	Gram (+) Pathogens	Yeast/Fungus	Main Results	Reference
-	0.034 μg Ag/mL	*Escherichia coli* K12	-	-	2 log reduction of *E. coli* after membrane filtration.	[29]
≈ 7 nm and 27.5 nm	0.26–26.5 mg Ag/dry g paper	*Escherichia coli*	*Enterococcus faecalis*	-	After filtration, the paper with a higher content of Ag-NPs almost completely deactivated bacterial growth. Reductions of 7 and 3 log were produced for *E. coli* and *E. faecalis*, respectively.	[30]
75 nm (spherical) and 8–20 nm (triangular)	-	*Escherichia coli, Pseudomonas aeruginosa, Salmonella typhi, Acinetobacter baumannii, Enterobacter cloacae, Haemophilus influenzae, Klebsiella pneumoniae, Neisseria mucosa, Proteus mirabilis, Serratia odorifera, Vibrio parahaemolyticus* and *Paenibacillus koreensis*	*Staphylococcus aureus, Bacillus subtilis* and *Paenibacillus koreensis*	*-*	The highest antimicrobial activity of the Ag-NPs was against *E. coli* and *P. aeruginosa*. For *S. typhi* and *B. subtilis* this activity was moderate and low for *S. aureus*.	[26]
14.6 nm	0.2, 0.5, 1, 1.5, 2 mg/mL	*Escherichia coli, Pseudomonas aeruginosa* and *Klebsiella pneumoniae*	*Lactobacillus rhamnosus* GG, *Bacillus cereus* and *Listeria monocytogenes*	*Aspergillus* and *Penicillium*	Inhibition of bacterial growth was dose dependent. *P. aeruginosa* was the bacteria most sensitive to Ag-NPs, followed by *E. coli*. On the contrary *L. monocytogenes* was the most resistant.	[31]
10, 20, 40, 60 and 80 nm	8 µg Ag/mL (10 nm), 11 µg Ag/mL (20 nm), 5 µg Ag/mL (40, 60 and 80 nm)	*Escherichia coli* and *Pseudomonas fluorescens*	*-*	*Saccharomyces cerevisiae*	Nanoparticles of a size equal to or less than 10 nm were more bioavailable when interacting with the cells. It was also shown that the toxicity of Ag-NPs decreased with increasing size.	[21]
8 nm (59 and 83 nm hydrodynamic size)	0–400 µg Ag/mL	*Proteus vulgaris* and *Shigella sonnei*	*Staphylococcus aureus, Bacillus megaterium*	-	The smaller size of Ag-NPs produced a greater growth inhibition. For both sizes the MIC values for the bacteria were between 75–400 ug/mL.	[20]
-	4.5 μg Ag/g film	*Pseudomonas* and *Enterobacteriaceae*	-	-	No significant differences were observed in the use of the film with nanoparticles compared to the conventional film.	[32]
10–50 nm	197 µg Ag/mL	*Campylobacter jejuni* (collection strain and isolates of patients and food chain)	-	-	The concentrations between 9.85 and 39.4 μg/mL were bactericidal after 24 h of incubation. In addition, the lower concentrations (1.23 and 4.92 μg/mL) significantly inhibited the growth of the collection strain.	[33]
-	-	*Escherichia coli*	*Staphylococcus aureus*	*Aspergillus niger* and *Penicillium citrinum*	The antimicrobial activity of the chitosan, laponite and Ag-NPs hybrid film turned out to be slightly less than the chitosan film because laponite decreases the release of silver. There was also a greater inhibition of gram-positive bacteria compared to gram-negative bacteria.	[34]
20–30 nm	2.37, 4.75, 9.5 and 19 μg Ag/mL	*Escherichia coli* and *Salmonella typhimurium*	*-*	*-*	The concentration of 4.75 μg/mL Ag-NPs completely inhibited the growth of the two bacteria and the concentration of 9.5 μg/mL was sufficient to kill them.	[35]
47.3 nm	0–100 μg Ag/mL	*Escherichia coli* O157:H7, *Vibrio parahaemolyticu*, *Pseudomonas aeruginosa* and *Salmonella typhimurium*	*Listeria monocytogenes* and *Staphylococcus aureus*	-	Ag-NPs exerted a strong antimicrobial activity against all the pathogens tested. MIC of *V. parahaemolyticus* and *S. aureus* were 6.25 μg/mL and 50 μg/mL, respectively, and MBCs of *V. parahaemolyticus* and *S. aureus* were 12.5 μg/mL and 100 μg/mL, respectively.	[36]
6–25 nm (chemical synthesis) 80–120 nm and 40–100 nm (synthesized with *Fusarium nivale* and *Penicillium glabrum*)	170 µg Ag/mL	*Pseudomonas aeruginosa* PA01 4/4–15	*Bacillus cereus* B 504T UNIQEM, *Staphylococcus aureus* 209p	*Fusarium oxysporum*	Chemically synthesized AG-NPs inhibited microbial growth at 6 h of exposure, while with microbiologically synthesized nanoparticles it occurred at 24 h. *S. aureus* was the most resistant microorganism to both types of Ag-NPs.	[12]
5–15 nm	0.5, 1.0, 2.5, 5.0, 7.5, 10.0, 20.0 and 30.0 μg Ag/mL	*Escherichia coli*	*Staphylococcus aureus* and methicillin-resistant *Staphylococcus aureus*	*-*	The nanoparticles produced a total inhibition of *E. coli* growth at the concentration of 7.5 μg/mL. On the contrary, a concentration of >30 μg/mL is required for the complete inhibition of *S. aureus* and the resistant strain.	[15]
10–20 nm	8.34 × 10^−7^, 3.61 × 10^−6^, 5.79 × 10^−5^ and 4.63 × 10^−4^ mol/L	*Escherichia coli*	*Staphylococcus aureus*	*-*	Ag-NPs exerted a higher antimicrobial activity than the AgNO3 solution. This activity was concentration dependent and greater than other studies in which they use green synthesis due to their small size and spherical shape.	[14]
-	-	*Salmonella typhimurium*	*Staphylococcus aureus*	-	The film that generated Ag-NPs in situ exerted a clear antimicrobial activity against both pathogens. A lower microbial growth was also observed when using this material to store chicken sausages for 4 days at 4 °C compared to the traditional film.	[37]
8–15 nm	30, 75, 150, and 300 μg Ag/mL	*Escherichia coli* O157:H7	*Listeria monocytogenes*	*-*	The material containing Ag-NPs exerted a greater antimicrobial activity against *E. coli* than against *L. monocytogenes* due to the greater wall thickness of the gram-positive bacteria.	[38]

**Table 2:** Studies regarding silver nanoparticles cytotoxicity effects in several cell lines.

Cell Line	Ag-NPs Size	Main Results	Reference
Periodontal fibroblasts extracted from volunteers	<10 nm, 15–20 nm, and 80–100 nm	Small-sized Ag-NPs (<20 nm) increased cytotoxicity in cells in a dose and time dependent manner.	[80]
Human gingival fibroblast (CRL-2014)	2 nm	Ag-NPs increased oxidative stress, inflammation and cell apoptosis.	[78]
Human gingival fibroblasts (HGF-1)	10 nm	All the nanoparticles tested were less toxic and exerted a greater antimicrobial action than the silver nitrate solution.	[79]
Human periodontal fibroblasts (HPLF)	-	Ag-NPs at low concentration did not alter morphology or cell proliferation, while at high concentration they significantly altered morphology, inhibited proliferation, and stopped cell cycle.	[81]
Human colon epithelial cells (Caco-2)	-	There were no significant differences in cell viability between digested and undigested nanoparticles up to a concentration of 40 μg/mL. There was a viability reduction (65%) when adding a food matrix.	[84]
EpiIntestinal, EpiOral and EpiGinvival tissues	16 nm in average with sporadic occurrence of particles with a size of around 80 nm	Ag-NPs did not affect the viability of EpiOral and EpiGingival tissues. In addition, the release of IL-1 decreased significantly in EpiOral tissue. On the other hand, exposure of the EpiIntestinal tissue to gastric fluids with or without AG-NPs produced a slight decrease in viability.	[85]
Human colon epithelial cells (HT-28 and HCT-116)	6 nm	After 24 h of exposure with Ag-NPS, a decrease in dose-dependent cell viability was observed (2–10 µg/mL). A cytotoxicity of approximately 50% was reached at a concentration of 4 µg/mL.	[86]
Human colon epithelial cells (HT-29 and Caco-2) and colon regular cells (CCD-18)	10–50 nm	Cytotoxicity occurred in the cells at a concentration of Ag-NPs between 9.85 and 39.4 μg/mL.	[33]
Human colon epithelial cells (Caco-2)	≈7.74 nm	In this work, there was no significant decrease in cell viability after 24 h at a concentration of 100 μg/mL.	[87]
Human colon epithelial cells (Caco-2/HT-29-MTX)	51–52 nm	Cellular uptake decreased when using digested versus undigested Ag-NPs and the nanoparticles coated with lipolic acid dissolved to a greater extent than those coated with citrate.	[88]
Human colon epithelial cells (Caco-2)	5–25 nm for PEG-AgNPs 20; 4–6 nm and 10–50 nm for GSH-AgNPs	A significant decrease in cell viability was observed by exposing cells to digested nanoparticles (both coatings), but not to undigested nanoparticles.	[74]
Rat brain microvessel endothelial cells (rBMEC)	25, 50 and 80 nm	Ag-NPs were more cytotoxic at lower concentrations for a size of 25 and 40 nm. On the contrary, for a size of 80 nm greater concentrations were needed.	[89]
Human breast epithelial cells (MCF-7)	20–80 nm	Ag-NPs caused apoptosis and necrosis in a dose-dependent manner to a concentration of 80 μg/mL. At higher concentrations, the apoptotic effect decreased while the necrotic effect became prominent.	[90]
Human liver epithelial cells (HepG2)	10 and 100 nm	Ag-NPs at low doses increased cell proliferation.	[91]
Human breast epithelial cells (MCF-7)	31.4 nm	Ag-NPs at a concentration of 60 µg/mL exhibited a cytotoxicity of 70% against the cell line. It was also observed that AgNP were much less cytotoxic when tested against a non-cancerous cell line.	[92]
Human dermal fibroblast (NHDF)	20–45 nm	Except for the sodium oleate and sodium dodecyl sulfate solutions, the rest prevented the aggregation of the nanoparticles, stabilized them and did not produce a significant cytotoxic effect on the cells.	[76]

**Table 3:** Studies in *in vitro* static and gastrointestinal simulation models regarding silver nanoparticles effects at gut level and microbiota.

Static/Dynamic	Particle Size	Main Results	Reference
Static	Ag-NPs 10–50 nm	The range of MIC and MBC for oral bacteria was between 100 and 250 µg/mL. Of the four oral bacteria tested, the most sensitive to silver nanoparticles were *Porphyromonas gingivalis* and *Fusobacterium nucleatum*.	[83]
Static	Ag-NPs 5, 15 and 55 nm	In this work it was observed that for the smaller nanoparticles the MIC was between 25 and 50 µg/mL. Oral aerobic bacteria were more susceptible than anaerobic bacteria.	[19]
Static	Ag-NPs 30–50 nm	A MIC between 15 and 90 µg/mL was reported for the exposure of Ag-NPs against 5 oral pathogens, much lower than for chlorhexidine.	[82]
Static	Ag-NPs 60 nm	AG-NPs of a size of 60 nm digested under physiological conditions can reach the wall of the intestine. It was also observed that after ingestion of Ag + ions nanoparticles ended up forming.	[71]
Static	Ag-NPs 10 and 75 nm	After the intake of Ag-NPs, these nanoparticles can be aggregated and chemically modified in the stomach depending on the size and surface chemistries.	[96]
Static	Ag-NPs 10 nm	There was a reduction in the production of capric and stearic fatty acids after exposure of the human feces sample to Ag-NPs, while palmitic acid increased. The presence of *Bacteroidetes* was also drastically reduced.	[99]
Static	16 nm in average with sporadic occurrence of particles with a size of around 80 nm	The size and morphology of the Ag-NPs changed due to the action of different gastric fluids and digestive enzymes. The study showed that nanoparticles agglomerate and partially react to form AgCl during exposure to fluids.	[85]
Static	Ag-NPs 14 nm	A decrease in *Bacteroidetes* and an increase in *Firmicutes* was observed, resulting in an alteration of the *Firmicutes*/*Bacteroidetes* ratio. Exposure with Ag-NPs for 24 h also altered the *Faecalibacterium prausnitzii* and *Clostridium coccoides*/*Eubacterium rectal* taxa.	[100]
Static	5–25 nm for PEG-AgNPs 20; 4–6 nm and 10–50 nm for GSH-AgNPs	AgNPs agglomerated less and were less toxic in colon cells than PEG-AgNPs 20.	[74]
Dynamic	Ag-NPs 15 and 40 nm	It was observed that 90% of the silver nanoparticles had dissolved as they passed through the stomach and the resulting ions joined the digestive matrices.	[73]
Dynamic SIMulator of the GastroIntestinal tract (simgi^®^)	3–5 nm and 10–25 nm for PEG-AgNPs 20; 4–6 nm and 10–50 nm for GSH-AgNPs	Ingestion of Ag-NPs did not alter the microbial composition of the intestine or the metabolic activity of the bacteria. It was also observed how during the digestion the nanoparticle size was predominantly 3–5 nm, although small populations of agglomerates of these small nanoparticles were found.	[98]

**Table 4:** In vivo studies regarding silver nanoparticles effects at gut level and microbiota, organs and tissues.

Model	Study Design	Main Results	Reference
C57BL/6N mice	Ag-NPs 29,3 nm Dose: 100 mg/kg, 500 mg/kg or 1000 mg/kg	The production of significant alterations of selective genes in the caudate, frontal cortex and hippocampus of mice was observed after exposure to the nanoparticles. The data concluded that nanoparticles can produce neurotoxicity by generating oxidative stress.	[101]
Sprague–Dawley rats	Ag-NPs 60 nm; 28 days Four groups (10 rats in each group): vehicle control, low-dose group (30 mg/kg), middle-dose group (300 mg/kg), and high-dose group (1000 mg/kg)	A dose-dependent increased accumulation of Ag-NPs was observed in the lamina propria in both the small and large intestine, and also in the tip of the upper villi in the ileum and protruding surface of the fold in the colon. Rats that consumed nanoparticles also released more anormal mucus in the crypt lumen and ileal lumen and there was also detachment of cells at the tip of the villi.	[102]
F344 rats	Ag-NPs 56 nm; 13 weeks Four groups (10 rats in each group): vehicle control, low-dose (30 mg/kg), middle-dose (125 mg/kg), and high-dose (500 mg/kg).	Significant dose-dependent changes were found in alkaline phosphatase and cholesterol, indicating that exposure to more than 125 mg/kg of silver nanoparticles may result in slight liver damage. Histopathologic examination revealed a higher incidence of bile-duct hyperplasia, with or without necrosis. There was also a dose-dependent accumulation of silver in all tissues examined.	[103]
Mice	Ag-NPs 3–20 nm; 21 days Daily dose: 5, 10, 15 y 20 mg/kg	Mice treated with a dose of 10 mg/kg showed great weight loss. It was found that Ag-NPs damaged the microvilli of epithelial cells and intestinal glands. This may be the cause of weight loss due to intestinal malabsorption.	[104]
Wistar rats	Ag-NPs 10 nm; 14 days Daily dose: 0.02 mg/kg	Ag-NPs intake produced a synaptic degeneration and potential neuronal cell death due to alterations in synaptic structures and reduced levels of proteins associated with these structures	[105]
Sprague–Dawley rats	Ag-NPs 3–10 nm (98.7%), 10–30 nm (1.3%); 14 days Daily dose: 1 mg/kg or 10 mg/kg Three groups (6 rats in each group): control group, low-dose group (1 mg/kg), high-dose group (10 mg/kg)	After ingestion of Ag-NPs, neuron shrinkage, cytoplasmic or foot inflammation of the astrocytes and extravascular lymphocytes occurred. This led to the conclusion that Ag-NPs can induce neuronal degeneration and swelling of astrocytes even with oral exposure at low doses.	[106]
C57BL/6NCrl mice	Ag-NPs 110 nm and 20 nm (PVP), 110 nm and 20 nm (Citrate); 28 days Daily dose: 10 mg/kg	None of the nanoparticles tested caused alterations in the structure or diversity of the intestinal microbiota of the mice.	[107]
Sprague–Dawley rats	Ag-NPs 10, 75 and 100 nm; 13 weeks Daily dose: 9, 18 and 36 mg/kg twice a day	It was possible to observe how the nanoparticles produced changes in the intestinal microbiota of the rats. There was an increase in Gram-negative bacteria. Exposure to smaller Ag-NPs resulted in a decrease in Lactobacillus spp. and the Firmicutes phyla.	[108]
Sprague–Dawley rats	Ag-NPs 12 nm; single exposure and multiple exposures over 30 days Daily doses: 2000 and 250 mg/kg for single and multiple administrations, respectively.	Single and multiple administrations resulted in silver accumulation in the liver, kidneys, spleen, stomach, and small intestine. But, concentrations of silver detected in tissues were far smaller than the administered doses (<99%), indicating its efficient excretion from the organism.	[109]
BALB/C mice	Ag-NPs 294 nm (NanoAg1) and 122 nm (NanoAg 2); 3 days Daily dose: 100 µL suspension	The administration of NanoAg1 increased the number of Clostridium perfringens and Escherichia coli and decreased that of Lactobacillus spp., But the results were not significant. NanoAg2 acted in reverse. It could also be seen how nanoparticle suspensions reversed a severe colonic lesion in mice.	[110]
Mice	Ag-NPs 55.17 nm; 28 days Doses: 0 (control), 11.4, 114 and 1140 μg Ag-NP/kg	In this work, an increase in the Firmicutes/Bacteroidetes ratio was observed, similar to that described in studies of obesity and inflammatory diseases.	[111]
Fish (*Piaractus mesopotamicus*)	Ag-NPs 50 nm; 24 h Dose: 0 (control), 2.5, 10, and 25 μg Ag-NPs/L	More silver accumulated in the brain than in gills and liver at all concentrations. There was also an increase in oxidative stress, as well as damage to the enterocytes in fish exposed to higher concentrations.	[112]
Zebrafish	Ag-NPs 58.6 nm; 14 days Dose: 500 mg/kg twice a day	Despite not finding lesions in the integrity of the intestinal epithelium, in this study it was observed that Ag-NPs decreased to a non-detectable level to beneficial bacterial populations of fish.	[113]
Zebrafish	Ag-NPs 10, 40 and 100 nm; 4 days Dose: 1, 5, 10, 50, 100, 150 y 200 ppm	It was observed that the salts and cations of the medium decreased the dissolution of the silver, thus limiting its action. Ag-NPs with a size of 10 and 100 nm caused developmental defects in the muscles and intestine of the embryo, while those of 40 nm produced lethal effects.	[114]
Zebrafish	Ag-NPs 20 and 100 nm; 96 h Dose: 0.61, 1.07, 0.67, and 1.28 mg/L	The coating of the nanoparticles increased the survival rate of the fish compared to the control. It was also observed that the smaller Ag-NPs were more lethal than the 100 nm. More nanoparticles accumulated in the intestines than in the gills.	[115]
*Caenorhabditis elegans*	Ag-NPs 79 nm	The effect of silver nanoparticles for 10 generations of the nematode was studied. From the second a pronounced sensitization to the nanomaterial was observed.	[116]
*Caenorhabditis elegans*	Ag-NPs 25 and 75 nm; 12 h Dose: 5 mg/L	Exposure of E. coli to the nanoparticles and of the nematode to E. coli induced reproductive toxicity, as well as neurotoxicity.	[117]
*Caenorhabditis elegans*	Ag-NPs <100; 40 h Dose: 0, 1, 3, and 5 mg/kg	Different silver nanomaterials induce growth inhibition and reproductive toxicity when the soil is found at a concentration of ≥5 mg/kg.	[118]
*Caenorhabditis elegans*	Ag-NPs ≈ 69 nm	Factors that increased sensitivity and reproductive toxicity from the second generation could not be verified. Therefore, long-term risk cannot be assessed and other inheritance mechanisms, such as epigenetics, may be at play in multigenerational reproductive toxicity.	[119]
*Drosophila melanogaster*	Dose: 10–100 µg Ag/mL (accute intake) and 5 µg Ag/mL (chronic exposure)	After the acute intake, a significant toxic effect was observed at the concentration of 20 µg/mL and 50% of the flies could not complete their development cycle. In the case of the chronic exposure in 8 generations, a decrease in fertility was observed in the first three generations, after which it returned to normal.	[120]
*Drosophila melanogaster*	Ag-NPs 5–22 nm Dose: 10, 50, 100, 200 g/mL	All nanoparticles tested (synthesized from different natural extracts) significantly reduced the number of hatched larvae. In addition, those synthesized from mulberry, fig and olive produced a high mortality of larvae and adults.	[121]
*Drosophila melanogaster*	Ag-NPs 20–100 nm; 3, 10 and 30 days Dose: 5, 25, 50 and 250 µg Ag/mL	The effect of Ag-NPs depends on the dose and the stage of development of the flies. In general it alters the ability to lay eggs, decrease the size of the ovary and decrease survival and longevity.	[122]
*Drosophila melanogaster*	Ag-NPs 3.44 nm; 10 days Dose: 0.016, 0.08, 0.4, 1 y 2 mM	The 10 nM dose was completely toxic. Despite this, depigmentation was observed at all concentrations. Significant levels of intracellular ROS and DNA damage were also observed.	[123]
Humans	Volunteers: 60 Ag-NPs 5–10 nm (10 ppm) or 25–40 nm (32 ppm) Study 1: 10 ppm with 3, 7, and 14 day time periods Study 2: 32 ppm for 14 days Daily dose: 100 μg/day for 10 ppm, and 480 μg/day for 32 ppm	No significant changes were observed in metabolism, hematology, urine, physical findings, sputum morphology or changes in images. Nor were statistically significant changes detected in the markers of hydrogen peroxide production or peroxiredoxin protein expression. Instead, silver could be detected in human serum.	[124]
